# Circulating microRNAs Correlate with Multiple Myeloma and Skeletal Osteolytic Lesions

**DOI:** 10.3390/cancers13215258

**Published:** 2021-10-20

**Authors:** Sara Reis Moura, Hugo Abreu, Carla Cunha, Cláudia Ribeiro-Machado, Carla Oliveira, Mario Adolfo Barbosa, Herlander Marques, Maria Inês Almeida

**Affiliations:** 1 Instituto de Investigação e Inovação em Saúde (i3S), University of Porto, 4200-135 Porto, Portugal; sara.moura@i3s.up.pt (S.R.M.); hugo.abreu@uniupo.it (H.A.); carla.cunha@ineb.up.pt (C.C.); claudia.machado@i3s.up.pt (C.R.-M.); coliveir@ineb.up.pt (C.O.); mbarbosa@i3s.up.pt (M.A.B.); 2 Instituto de Engenharia Biomédica (INEB), University of Porto, 4200-135 Porto, Portugal; 3 Instituto de Ciências Biomédicas Abel Salazar (ICBAS), University of Porto, 4050-313 Porto, Portugal; 4 Hospital de Braga, 4710-243 Braga, Portugal; herlander.marques@hb.min-saude.pt; 5 Centro Clínico Académico 2CABraga/ICVS, University of Minho, 4710-243 Braga, Portugal; 6 Centro de Investigação em Tecnologias e Serviços de Saúde (CINTESIS), Faculty of Medicine, University of Porto, 4169-007 Porto, Portugal

**Keywords:** diagnostic, biomarkers, non-coding RNAs, plasma, cancer, vertebrae

## Abstract

**Simple Summary:**

Molecular biomarkers for the diagnosis of multiple myeloma and for the early detection of the associated osteolytic lesions are needed. MicroRNAs are a class of small non-coding RNAs that regulate gene expression post-transcriptionally and have been explored as circulating (extracellular) biomarkers for distinct diseases. Results show that miR-16-5p, miR-20a-5p, and miR-21-5p levels are differently expressed in the plasma of multiple myeloma patients compared with the control group and suggest that their combined expression could be used as a potential circulating biomarker. Furthermore, the expression of plasma microRNAs significantly correlates with myeloma bone disease and with bone lesions in the spine.

**Abstract:**

Multiple myeloma (MM) is the second most frequent hematological disease and can cause skeletal osteolytic lesions. This study aims to evaluate the expression of circulating microRNAs (miRNAs) in MM patients and to correlate those levels with clinicopathological features, including bone lesions. A panel of miRNAs associated with MM onset and progression, or with bone remodeling, was analyzed in the plasma of 82 subjects (47 MM patients; 35 healthy controls). Results show that miR-16-5p, miR-20a-5p, and miR-21-5p are differently expressed between MM patients and healthy controls. Receiver operating characteristic analyses indicate that their combined expression has potential as a molecular marker (Area Under the Curve, AUC of 0.8249). Furthermore, significant correlations were found between the analyzed miRNAs and disease stage, treatment, β2 microglobulin, serum albumin and creatinine levels, but not with calcium levels or genetic alterations. In this cohort, 65.96% of MM patients had bone lesions, the majority of which were in the vertebrae. Additionally, miR-29c-3p was decreased in patients with osteolytic lesions compared with patients without bone disease. Interestingly, circulating levels of miR-29b-3p correlated with cervical and thoracic vertebral lesions, while miR-195-5p correlated with thoracic lesions. Our findings suggest circulating miRNAs can be promising biomarkers for MM diagnosis and that their levels correlate with myeloma bone disease and osteolytic lesions.

## 1. Introduction

Multiple myeloma (MM) is a malignant hematological disease characterized by excessive proliferation of abnormal plasma cells (PCs) in the bone marrow [1]. It accounts for more than 10% of all hematologic cancers and it remains incurable in the majority of cases, with a 45% 5-year survival rate [2,3]. Up to 80% of the newly diagnosed MM patients have osteolytic lesions that develop as a consequence of the exacerbated proliferation of malignant PCs in the bone marrow, causing an unbalance between the activity of bone-forming cells (osteoblasts) and bone-resorbing cells (osteoclasts) [1,4]. This leads to the disruption of the normal bone remodeling process and to the development of myeloma bone disease (MBD) [5,6]. Consequently, MM patients are at high risk of skeletal-related incidents such as pathological fractures, spinal cord compression, vertebral collapse, severe bone pain and hypercalcemia [7]. Notably, the spine is the most frequent location of the bone lesions caused by MM and vertebral crush fractures are common [8]. Bisphosphonates are pyrophosphate analogues that cause apoptosis of osteoclasts and are currently the gold standard pharmacological approach to prevent and treat MBD [7,9]. More recently, denosumab, a humanized monoclonal antibody against RANKL, has also been used to decrease osteoclastogenesis and resorption by osteoclasts [7]. Imaging tools, such as whole-body low-dose computed tomography (CT), positron emission tomography-CT, and whole-body magnetic resonance imaging, are used to detect osteolytic lesions [10], and there are no currently available molecular biomarkers to detect MBD. Therefore, new tools for the early diagnosis of MM, as well as for the rapid identification of MBD, are needed. This will contribute to initiate the treatment at an early stage, avoiding further complications.

MicroRNAs (miRNAs) are a class of small RNA molecules that is transcribed from the genome but do not encode proteins [11]. In their mature form, miRNAs are about 19–22 nucleotides in length and are able to regulate the expression of messenger RNAs [11] and long non-coding RNAs [12]. Importantly, miRNA expression is disrupted in a vast number of diseases and hematological malignancies, such as MM [13,14]. Specifically, miRNAs are involved in MM onset and progression, which triggered their study as therapeutic targets [14]. Since the last two decades, circulating (extracellular) miRNAs have emerged as potential biomarkers for a wide range of diseases [11,15], including for MM [13]. Considering miRNAs can be detected in human blood serum/plasma, urine, saliva and other body fluids [11], these molecules are promising diagnosis tools through non-invasive or minimally invasive methods [15], leading to the development of several miRNA-based products for disease diagnosis. The stability of endogenous miRNAs in plasma samples is an important prerequisite for biomarkers [16]. It has been shown that miRNAs can be packed into extracellular vesicles [17] or can be associated with protein complexes [18], namely AGO proteins, protecting them from degradation by nucleases. Still, one of the main current challenges is to increase the sensibility and specificity of these circulating biomarkers [15].

The aim of this study is to identify circulating (extracellular) miRNAs to be used as biomarkers for the diagnosis of MM or MBD. For this purpose, a panel of miRNAs associated with MM onset and progression or with MBD pathophysiology, including regulation of osteoblasts and osteoclasts-related mechanisms, was evaluated. Levels of circulating miRNAs were analyzed in plasma samples and correlated with MM clinico-pathological characteristics and with bone lesions, including in the spine. Results show that circulating miRNAs are promising candidates for the diagnosis of MM and for the MM-associated skeletal osteolytic lesions.

## 2. Materials and Methods

### 2.1. Patients and Plasma Samples

Human peripheral blood from patients diagnosed with MM (*n* = 47) was collected at Oncology Department at Hospital de Braga (Braga, Portugal) between July 2018 and January 2021. Patients with other tumor types or inflammatory diseases were excluded from the study. Peripheral blood from healthy donors (*n* = 35) was collected at Immunohemotherapy Department of Centro Hospitalar de São João (CHSJ, Porto, Portugal). The blood donors used in this study do not have history of cancer or inflammatory diseases, had not been diagnosed with infectious diseases and were not under the effect of anti-inflammatory or anti-depressive drugs. A written informed consent was obtained from all subjects before blood collection. The patients’ clinical data was pseudonymized. The study was approved by Hospital de Braga and CHSJ Ethics Committee for Health, and the protocol conforms to the declaration of Helsinki. Clinical data was collected from medical records. Stage of the disease was determined following the International Staging System (ISS) for multiple myeloma, genetic alterations were assessed by cytogenetics/fluorescence in situ hybridization (FISH), and bone osteolytic lesions were assessed by magnetic resonance imaging, computerized tomography or conventional X-ray.

The blood samples were transferred from the EDTA collection tubes to 15 mL tubes, centrifuged at 1200× *g*, for 20 min, at room temperature (RT), and the plasma was collected. All samples were processed within three hours following blood collection to prevent hemolysis. No signs of colors from faint pink to bright red were detected in plasma samples following blood centrifugation. Plasma was further centrifuged at 2500× *g*, for 10 min, at 4 °C, to remove cell debris and stored at −80 °C in microtubes until further use.

### 2.2. Isolation and Purification of Circulating microRNAs

MiRNAs present in plasma samples were isolated and purified with miRNeasy Serum/Plasma Kit (Qiagen), according to the manufacturer’s instructions. Briefly, 1 mL of QIAzol Lysis Reagent was added to 200 µL of plasma. After vortexing and incubating at RT for 5 min, 2 µL (1.5 nM) of spike-in controls (cel-miR-39-3p and cel-miR-54-3p) were added. Then, 200 µL of chloroform was added, samples vigorously mixed, centrifuged at 12,000× *g*, for 15 min, at 4 °C, and the upper aqueous phase was collected to a new tube, with addition of 900 µL of ice-cold pure ethanol. Samples were transferred to the RNeasy MinElute spin columns, washed, and RNA was eluted in 15 µL of RNase-free water. Finally, RNA concentration was determined by measuring the absorbance in a NanoDrop Spectrophotometer ND-1000 (ThermoFisher Scientific, CA, USA).

### 2.3. Selection of the miRNA Panel, Reverse Transcription and Real Time Quantitative Polymerase Chain Reaction (RT-qPCR)

The expression levels of 10 miRNAs associated with MM onset and progression or with bone biological processes (miR-16-5p; miR-20a-5p; miR-21-5p; miR-29a-3p; miR-29b-3p; miR-29c-3p; miR-93-5p; miR-99a-5p; miR-146a-5p and miR-195-5p) were tested. In detail, selection of this panel was based on the following criteria: (1) miRNAs involvement in osteoblasts (bone forming cells) differentiation (miR-20a-5p, miR-29b-3p, miR-29c-3p, miR-146a-5p, miR-195-5p [19], miR-99a-5p [20], miR-93-5p [21], miR-29a-3p [22]), (2) miRNAs in multiple myeloma that mediate communication with cells in the tumor microenvironment (miR-16-5p [23], miR-21-5p [24]), (3) miRNAs dysregulated in MM cells or cell lines [25]. Importantly, several of the above mentioned miRNAs are concomitantly involved in two or more of these processes.

RNA was converted to cDNA through TaqMan^®^ Advanced miRNA cDNA Synthesis Kit (Applied Biosystems), according to the manufacturer´s protocol. Firstly, for the poly(A) tailing reaction, RNA samples were mixed with Poly(A) Buffer, ATP, Poly(A) Enzyme and RNase-free water, briefly vortexed and incubated in a thermocycler (MyCycler Thermo Cycler, Bio-Rad, Hercules, CA, USA) at 37 °C for 45 min followed by 10 min at 65 °C. For the adaptor ligation reaction, DNA Ligase Buffer, 50% PEG 8000, Ligation Adaptor, RNA Ligase and RNase-free water were mixed with the previous reaction and incubated at 16 °C for 1 h. Then, the reverse transcription was performed by adding RT Buffer, dNTP, Universal RT Primers, RT Enzyme Mix and RNase-free water and incubated at 42 °C for 15 min and 5 min at 85 °C. Finally, for cDNA amplification, miR-Amp Master Mix, miR-Amp Primer Mix and RNase-free water was added to 5 µL of the RT reaction product and incubated with the following settings: enzyme activation for 5 min, at 95 °C; denaturation at 95 °C for 3 s and annealing/extension at 60 °C for 30 s, for 14 cycles; stop reaction at 99 °C for 10 min. Then, cDNA was diluted in 50 µL of RNase-free water and stored at −20 °C until further use.

For the RT-qPCR reaction, cDNA was mixed with TaqMan Fast Advanced Master Mix (Applied Biosystems), TaqMan Advanced miRNA Assay for miR-16-5p, miR-20a-5p, miR-21-5p, miR-29a-3p, miR-29b-3p, miR-29c-3p, miR-93-5p, miR-99a-5p, miR-146a-5p or miR-195-5p, and nuclease-free water in a 7500 Fast Real-Time PCR System (Applied Biosystems) and incubated as follows: 95 °C for 20 s; 40 cycles of 95 °C for 3 s and 60 °C for 30 s. RT-qPCR reactions were performed in duplicates. The results were analyzed in the 7500 Software v2.0.6 (Applied Biosystems). Exogenous cel-miR-39-3p and cel-miR-54-3p were used as normalizers. The relative gene expression was calculated using the ΔCq method, in accordance with MIQE guidelines [26].

### 2.4. Statistical Analysis

Statistical data analysis was performed using the GraphPad Prism v8.1.2 software (GraphPad Software Inc, San Diego, CA, USA) and SPSS Statistics v26 (IBM Corp, Armonk, NY, USA). D’Agostino and Pearson omnibus normality tests were performed to evaluate if data followed a Gaussian distribution. Considering that data did not follow a normal distribution, the non-parametric Mann–Whitney U test was used to evaluate significant differences between groups. Categorical data was tested using the Fisher’s exact test. 

Relative expression of miRNAs was compared across groups using a linear regression, adjusted for the baseline characteristic identified as a possible confounder (age). The diagnostic value of the circulating miRNAs in plasma samples was calculated using Receiver Operating Characteristics (ROC) curve. To obtain the value of combined miRNAs expression a binary logistic regression was performed, considering the disease as the dependent variable and the miRNAs levels as covariates. For the ROC curve, the *p*-value tests the null hypothesis that the area under the curve equals 0.5. The cut-off points with the highest sensitivity and specificity were determined. Considering the data follows a non-parametric distribution, Spearman’s rank correlation was used to calculate correlation coefficient and significance between miRNA expression and clinicopathological parameters. Recursive partitioning (RP) was used to split the dataset iteratively based on certain statistical criteria. The RP classification trees were performed using rpart package from R software and Gini index criteria. Statistical significance was considered for *p* < 0.05.

## 3. Results

### 3.1. Patients Characteristics

Demographic, clinical and biochemical characteristics, treatment regimen, and genetic alterations in the patients’ cohort are shown in Table 1. Patients with MM were significantly older than healthy donors (*p* < 0.001), but no differences were found between groups regarding gender. The majority of the MM patients (53%) are at stage II, according to the International Staging System (ISS). In addition, IgG was the immunoglobulin most commonly produced by these patients (70%). Genetic alterations are present in MM patients, mainly P53 and RB1 deletion in 23% and 35% of the patients, respectively (Table 1).

### 3.2. Differences in the Expression of Circulating miRNAs between Multiple Myeloma Patients and Control Group

**Figure 1:**
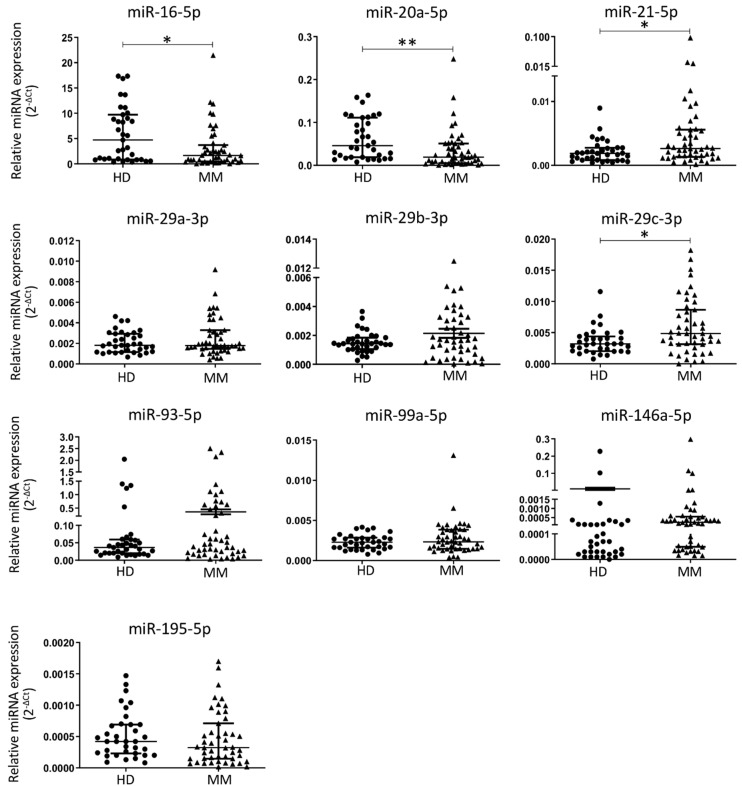
Expression levels of circulating microRNAs in plasma samples from multiple myeloma patients (MM, *n* = 47) and healthy donors (HD, *n* = 35) (median, interquartile range; Mann–Whitney test, * *p* < 0.05 and ** *p* < 0.01).

The expression of the selected 10 miRNAs was evaluated in the plasma of 47 MM patients and 35 healthy donors. Results show that miR-16-5p and miR-20a-5p were significantly downregulated, while levels of miR-21-5p and miR-29c-3p were significantly upregulated, in MM patients compared with the control group (Figure 1). Other members of the miR-29 family, namely miR-29a-3p and miR-29b-3p, were not statistically different between groups (Figure 1). Levels of four additional miRNAs, namely miR-93-3p, miR-99a-5p, miR-146a-5p and miR-195-5p, were also tested but no statistically significant differences were found between groups (Figure 1). Notably, miR-16-5p and miR-93-3p were among the most highly expressed miRNAs in plasma samples with median CT values of 13.96 and 19.84 cycles, respectively. However, miR-93-3p shows high variability, both in the MM and in the control group. On the other hand, miR-146a-5p was the miRNA with the lowest expression in the plasma, with a median Ct value of 28.77 cycles. Importantly, all tested miRNAs show Ct values below the threshold of 35 cycles. 

**Figure 2:**
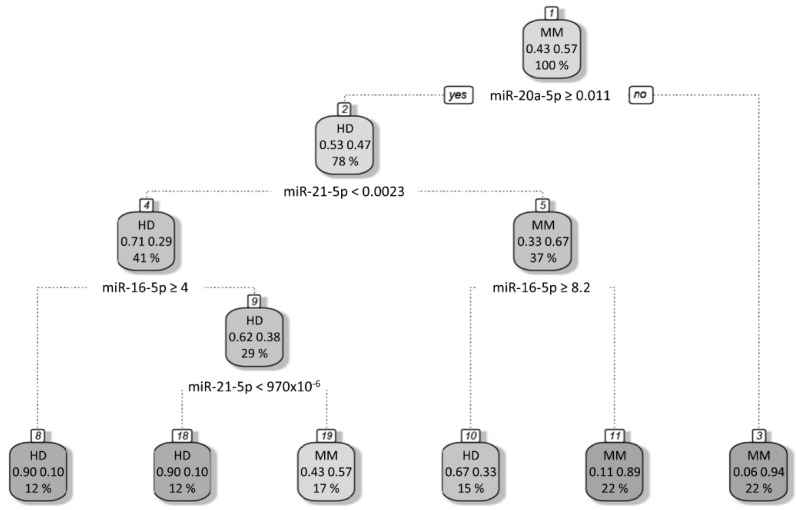
Decision tree created with 4 splits of the data. The root node (Node 1) contains all the data in the dataset. The algorithm splits the data based on a defined statistic, creating two new nodes (Node 2 and Node 3). Using the same statistic, it splits the data again at Node 2, creating two more leaf nodes (Nodes 4 and 5). For instance, Node 4 splits into Node 8 and 9; and Node 5 splits in two leaf nodes (Node 10 and 11). Lastly, it splits the data at Node 9, creating two more nodes (Nodes 18 and 19). The decision tree makes its prediction for each data row by traversing to the leaf nodes (one of the terminal nodes: Node 8, 18, 19, 10, 11 or 3).

Considering age as a variable that is significantly different between MM samples and the control group (Table 1), miRNAs miR-16-5p, miR-20a-5p, miR-21-5p and miR-29c-3p levels were tested again, adjusted for the age variable. The results from the multivariate linear regression analysis show that miR-16-5p, miR-20a-5p, and miR-21-5p maintain statistical significance, while miR-29c-3p differences could not be proven as age-independent (Table 2). Moreover, results from RP analysis show that the worst prognostic group refers to patients who have low expression of miR-20a-5p (miR-20a-5p < 0.011). The RP was subsequently used to maximize the variance across different nodes and minimize the variance within each node, allowing the identification of potential interactions between miRNAs biomarkers and MM disease. Using combined data, RP analysis identified different risk groups for MM disease (Figure 2). Only age-independent miRNAs differentially expressed between healthy individuals and MM patients were used for this analysis. The worst prognostic group referred to individuals who had low values of miR-20a-5p (94% out of miR-20a-5p < 0.011 individuals have MM disease). On the other hand, individuals with high levels of miR-20a-5p (miR-20a-5p ≥ 0.011), low levels of miR-21-5p (miR-21-5p < 0.0023) and high levels of miR-16-5p (miR-16-5p ≥ 4) or with high levels of miR-20a-5p (miR-20a-5p ≥ 0.011), low levels of miR-21-5p (miR-21-5p < 0.0023), low levels of miR-16-5p (miR-16-5p < 4) and low levels of miR-21-5p (miR-21-5p < 970 × 10^−6^) had the best prognosis (90% of these individuals are healthy) (Figure 2). Herein, among the tested miRNAs, miR-16-5p, miR-20a-5p, and miR-21-5p were identified as differently expressed in the plasma of MM patients compared with healthy donors.

### 3.3. Specificity and Sensibility of Circulting microRNAs as Multiple Myeloma Biomarkers

Next, attempting to find a miRNA or a combination of miRNAs that could successfully be used as a biomarker for MM, ROC analysis was performed for the miRNAs that were previously found differentially expressed between the MM group and the control group.

**Figure 3:**
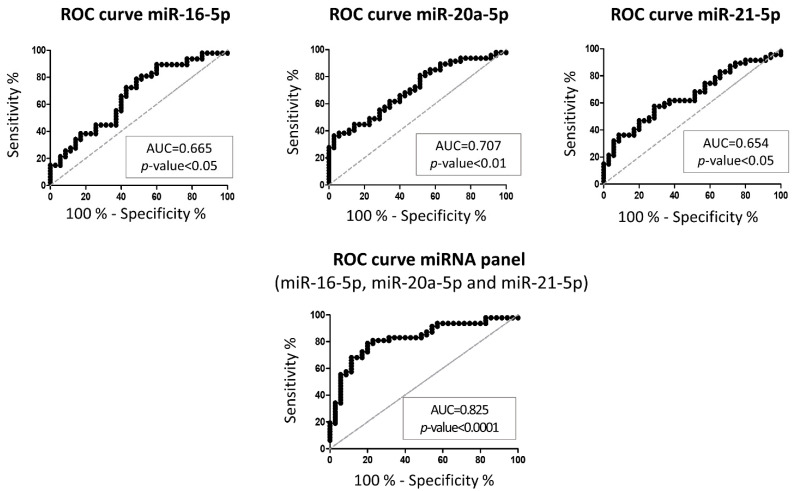
Receiver Operating Characteristic (ROC) curve of miR-16-5p, miR-20a-5p, miR-21-5p or combined panel of these 3 miRNAs between multiple myeloma patients and healthy controls (AUC: area under the ROC curve).

ROC curve analysis of miR-16-5p expression showed 78.72% sensitivity and 51.43% specificity in discriminating between MM and healthy donors, corresponding to an Area Under the Curve (AUC) of 0.665 (*p* < 0.05, 95% CI: 0.5458–0.7843). The miR-21-5p has an AUC of 0.654 (*p* < 0.05; sensitivity = 57.45%; specificity = 71.43%, 95% CI: 0.5365–0.7717). Considering the analysis of individual miRNAs, the highest AUC value was found for miR-20a-5p expression (0.707, *p* < 0.01; sensitivity = 36.17%; specificity = 97.14; 95% CI: 0.5961–0.8179). Nevertheless, combination of these three miRNAs improved the efficacy of the predictions, increasing both AUC and statistical significance. Specifically, combining miR-16-5p, miR-20a-5p and miR-21-5p improves AUC to 0.825 (*p* < 0.0001, sensitivity = 78.72%; specificity = 80.00%, 95% CI: 0.7333–0.9166) (Figure 3, Supplemental Table S1).

### 3.4. Correlation between Circulating microRNAs and Clinicopathological Variables in Multiple Myeloma Patients

Correlations of the miRNA levels in MM plasma samples with clinical and biochemical properties, treatment or chromosomal abnormalities was also tested. The miRNAs miR-20a-5p, miR-99a-5p and miR-195-5p negatively correlate with stage of the disease, following ISS classification (Table 3). Moreover, negative correlations were found between circulating miRNA levels and serum proteins. Among those, miR-16-5p, miR-20a-5p, miR-29b-3p, miR-29c-3p, miR-99a-5p and miR-195-5p were negatively correlated with β2M levels, a serum marker of tumor burden in hematologic malignancies [27,28], while miR-29a-3p was the only extracellular miRNA that negatively correlated with serum albumin (Table 3), a protein with prognostic value in MM patients [29,30]. Interestingly, besides miR-20a-5p, the three members of the miR-29 family showed a negative correlation with creatinine serum levels (Table 3), commonly used to measure the degree of insufficiency or renal failure [31]. No associations were found between the expression of circulating miRNAs and calcium levels (Table 3).

Regarding treatment of MM patients, a negative correlation was found between two members of the miR-29 family (namely miR-29b-3p and miR-29c-3p) and the use of chemotherapy or VTD regimen, while miR-93-5p and miR-146a-5p were negatively correlated with bone therapy (Table 4). Regarding genetic alterations, specifically del17p, del13q, translocation t(11;14), and translocation t(4;14), no significant correlations were found for any of the miRNAs (Table 4).

### 3.5. Correlation between Circulating microRNAs and Skeletal Osteolytic Lesions

**Figure 4:**
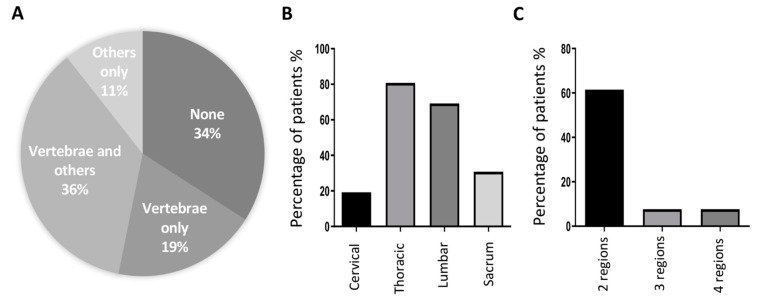

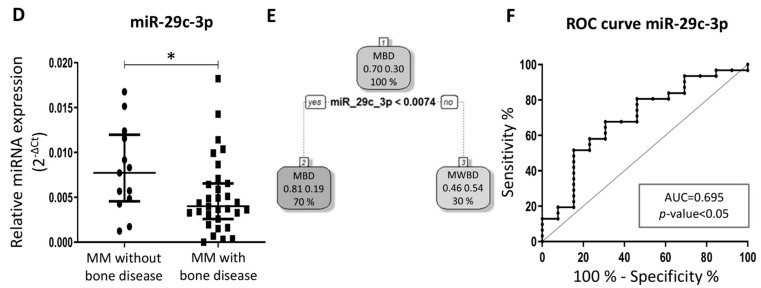
Bone lesions in multiple myeloma patients and miR-29c-3p expression. (**A**) Bone injury sites in multiple myeloma patients; (**B**) percentage of multiple myeloma patients with spinal lesions per region (cervical, thoracic, lumbar or sacrum); (**C**) percentage of multiple myeloma patients with spinal lesions in more than one region; (**D**) expression of circulating miR-29c-3p in multiple myeloma patients with and without bone lesions (median, interquartile range; Mann-Whitney test, * *p* < 0.05); (**E**) decision tree created with 1 split of the data. The root node (Node 1) contains all the data in the dataset. The algorithm splits the data based on a defined statistic, creating two new nodes (Node 2 and Node 3). MBD—myeloma bone disease, and MWBD—myeloma without bone disease; (**F**) Receiver Operating Characteristic curve of miR-29c-3p between patients with and without bone lesions (AUC: area under the ROC curve).

Thirty one, out of the 47 MM patients included in the cohort, have bone lesions (65.96%). Of those, 55% have osteolytic lesions in the vertebrae, with or without lesions in other bone regions of the skeleton (Figure 4A). The most affected regions are the thoracic (80.77%; T1-T12) and the lumbar vertebrae (69.23%; L1-L5), while sacrum and cervical lesions account for 30.77% and 19.23%, respectively (Figure 4B). Considering lesions in spinal regions, 61.52% of the patients had lesions in at least two spinal regions, while only 7.69% had injuries in all the regions (Figure 4C).

Our results show that circulating miR-29c-3p was the only miRNA significantly different between MM patients with and without bone lesions. Patients with bone lesions have a downregulation of about 36% in the expression of miR-29c-3p compared with patients without bone lesions, independent of the location of the lesions (Figure 4D, Supplemental Table S2). In agreement, when analyzing all the miRNAs of the panel by RP, only miRNA-29c-3p was identified and the results show that the worst prognostic group refers to patients who have low values of miR-29c-3p (miR-29c-3p < 0.0074) (Figure 4E). Importantly, age and gender are not significantly different between these two groups, miR-29c-3p being an independent marker. Furthermore, ROC curve analysis revealed that miR-29c-3p has discriminatory ability as diagnostic biomarker (AUC = 0.695; *p* < 0.05) (Figure 4F). In agreement, miR-29c-3p is statistically correlated with bone lesions (r = −0.308; *p* = 0.042) (Table 5). When considering lesions in specific spinal regions, miR-29b-3p correlates with cervical and thoracic vertebral lesions, while miR-195-5p correlates with thoracic lesions only (Table 5). No correlations were found between miRNAs expression and lesions in the lumbar or sacrum regions.

## 4. Discussion

Early detection of MM and of MM-related bone disease may lead to faster interventions and more effective treatments. Circulating miRNAs are excellent candidates to be used as biomarkers for the early diagnosis of diseases through non-invasive or minimally invasive methods [11]. In this study, plasma samples were screened for the expression of 10 miRNAs that had been selected due to their role in MM onset/progression or in the bone remodeling process. Herein, miR-16-5p, miR-20a-5p and miR-21-5p were identified as potential biomarkers for MM, particularly when combined together. Interestingly, Rocci et al. identified miR-16 and miR-21 as consistently expressed in at least 20% of MM samples, and further associated low miR-16 expression with poor overall survival [32], while Qu et al. showed miR-20a as downregulated in 33 MM patients versus 20 control samples in a Chinese cohort [33]. An advantage of using a panel of miRNAs compared with individual miRNA levels is that it increases accuracy and diagnosis value, as shown for other tumor types, including breast [34,35], lung [36], and bladder [37], among others.

Importantly, differences in circulating miRNAs between MM patients and healthy controls do not necessarily translate the differential expression found in the malignant PCs versus normal PCs. Interestingly, the secretion of miRNAs to the plasma may be caused by the tumor cells themselves, by the cells in the tumor microenvironment, including immune cells, or by cells that have their function indirectly dysregulated by the tumor. The miR-16-5p is a tumor suppressor downregulated in MM-PCs, while miR-21-5p is an oncogene overexpressed in malignant PCs [25]. The same expression profile was identified in our cohort of plasma/blood samples from MM patients, compared with healthy controls, for these two miRNAs. However, this is not the case for miR-20a-5p. In vitro and in vivo studies showed that miR-20a-5p has an oncogenic role, being upregulated in MM-PCs [38,39]. Nevertheless, levels of this transcript were found to be decreased in the plasma samples from MM patients, suggesting alterations in miR-20a-5p secretion to the plasma may not be directly related with the changes in the malignant cells themselves, but rather related with changes in cells from the tumor microenvironment. This is in line with previous findings that miR-20a-5p levels are decreased in both the bone marrow microenvironment and in the peripheral blood of MM patients [40].

In this study, the association between circulating miRNAs and osteolytic lesions was also investigated, with a particular focus in the spine. The extracellular levels of miR-29c-3p were decreased in MM patients with bone disease compared with those without bone lesions. Recently, Papanota et al. identified let-7b-5p, miR-143-3p, miR-17-5p, miR-214-3p and miR-335-5p as associated with osteolytic bone disease in MM. Both studies are complementary as they use distinct miRNA panels, and contribute to reach the goal of finding novel non-invasive markers for osteolytic lesions [41]. Interestingly, Kapinas et al. showed that miR-29c-3p is highly expressed in fully mature and mineralized mouse-derived osteoblasts [42]. This miRNA is a member of the miR-29 family, known to directly target 16 extracellular matrix genes [43], including genes expressed in the bone. Although miR-29c-3p correlates with bone disease, no significant correlations were found when considering the distinct spinal regions. On the other hand, miR-29b-3p negatively correlated with vertebral lesions in the cervical and thoracic regions. This miRNA was shown to promote osteoblasts differentiation at early stages by directly targeting inhibitors of osteogenesis, while it reduces collagen levels at late stages to facilitate maturation of the collagen fibrillar matrix and, consequently, mineral deposition [44]. Circulating levels of miR-29b-3p were previously found to correlate with histomorphometric parameters in the bone [45]. However, to the best of our knowledge, this is the first study correlating extracellular miRNA levels and osteolytic lesions in the spine caused by MM. 

## 5. Conclusions

Globally, we identified a promising combination of miRNAs in the plasma as biomarkers for MM. In addition, we specifically correlated circulating miRNA levels with the bone disease caused by MM, particularly with spinal osteolytic lesions. Future studies should validate these results in a larger multi-centric cohort of patients.

## Figures and Tables

**Table 1:** Characterization of multiple myeloma patients (MM) and healthy donors.

	Healthy Donors	MM	*p*-Value
**Demographic characteristics**			
Age (years)	39.00 (24–60)	71.00 (47–89)	<0.001
Gender (Male-Female) (%)	45.7–54.3	66.0–34.0	0.08
**Clinical and biochemical characteristics**			
ISS stage (I-II-III) (%)	N/A	23.53–52.94–23.53	
Ig isotype (IgA-IgD-IgE-IgG-IgM) (%)		23.91-0-0-69.57-0	
Light chains (%)		6.52	
β2-microglobulin (ng/mL)		4389(1386–29094)	
Albumin (g/dL)		3.35 (2.3–4.2)	
Creatinine (mg/dL)		1.1 (0.5–6.3)	
Calcium (mg/dL)		8.8 (6.4–11.8)	
**Treatment**			
Chemotherapy (%)	N/A	38.10	
Radiotherapy (%)		13.64	
Bone therapeutic (%)		28.57	
**Genetic alterations**			
del(17)(p13.1) (%)	N/A	23.26	
del(13)(q14.3) (%)		34.88	
t(11;14) (%)		13.64	
t(4;14) (%)		2.33	

For continuous variables: values represent median (min value–max value) and differences were assessed using non-parametric Mann-Whitney test; for categorial variables: values represent percentages and differences were assessed using Fisher's exact test; N/A: non-applicable.

**Table 2:** Statistical differences in microRNA levels between multiple myeloma patients and healthy donors, when adjusted to age.

miRNA	*p*-Value (Adjusted to Age)
miR-16-5p	0.027
miR-20a-5p	0.024
miR-21-5p	0.031
miR-29c-3p	0.334

**Table 3:** Correlation between circulating microRNAs and stage of the disease or serum protein levels in multiple myeloma patients. For each microRNA, the Spearman’s correlation coefficient (r) and significance level (*p*-value) are shown. Statistically significant correlations are highlighted in blue.

		Stage (ISS)			Serum Proteins			
		I	II	III	β2M (ng/mL)	Albumin (g/dL)	Creatinine (mg/dL)	Total Calcium (mg/dL)
**miR-16-5p**	r	0.155	0.102	−0.276	**−0.414**	−0.178	−0.287	0.053
	*p*	0.380	0.566	0.115	**0.013**	0.286	0.077	0.756
**miR-20a-5p**	r	0.283	0.078	**−0.375**	**−0.565**	−0.183	**−0.461**	0.043
	*p*	0.105	0.661	**0.029**	**0.000**	0.271	**0.003**	0.798
**miR-21-5p**	r	−0.170	0.096	0.057	0.146	−0.296	−0.150	−0.175
	*p*	0.338	0.589	0.751	0.402	0.071	0.362	0.300
**miR-29a-3p**	r	−0.007	0.156	−0.177	−0.315	**−0.349**	**−0.328**	0.042
	*p*	0.968	0.378	0.317	0.065	**0.032**	**0.042**	0.804
**miR-29b-3p**	r	0.233	0.066	−0.311	**−0.499**	−0.124	**−0.414**	0.054
	*p*	0.184	0.710	0.073	**0.002**	0.464	**0.010**	0.754
**miR-29c-3p**	r	0.198	−0.060	-0.127	**−0.419**	−0.157	**−0.326**	−0.060
	*p*	0.262	0.736	0.473	**0.012**	0.347	**0.043**	0.726
**miR-93-5p**	r	0.283	0.018	−0.304	−0.275	−0.041	−0.039	−0.123
	*p*	0.105	0.919	0.081	0.111	0.805	0.815	0.469
**miR-99a-5p**	r	0.000	0.312	**−0.368**	**−0.357**	−0.197	−0.007	0.013
	*p*	1.000	0.072	**0.032**	**0.035**	0.235	0.966	0.938
**miR-146a-5p**	r	−0.205	0.240	−0.078	0.082	−0.196	−0.015	−0.085
	*p*	0.245	0.171	0.662	0.642	0.239	0.927	0.618
**miR-195-5p**	r	0.254	0.078	**−0.346**	**−0.344**	−0.109	−0.238	−0.194
	*p*	0.146	0.661	**0.045**	**0.043**	0.513	0.144	0.250

**Table 4:** Correlation between circulating miRNAs and treatment regimens or chromosomal abnormalities in multiple myeloma patients. For each miRNA, the Spearman’s correlation coefficient (r) and significance level (*p*-value) are shown. Statistically significant correlations are highlighted in blue.

		Treatment			Genetic Alterations			
		Chemotherapy or VTd Regimen	Radiotherapy	Bone Therapy	del(17)(p13.1)	del(13)(q14.3)	t(11;14) Translocation	t(4;14) Translocation
**miR-16-5p**	r	−0.150	−0.162	0.216	0.253	0.016	−0.114	−0.114
	*p*	0.344	0.294	0.164	0.102	0.920	0.468	0.461
**miR-20a-5p**	r	−0.138	−0.089	0.114	0.271	−0.063	−0.081	−0.078
	*p*	0.385	0.567	0.466	0.079	0.689	0.605	0.615
**miR-21-5p**	r	−0.121	−0.063	−0.155	−0.200	−0.094	0.027	−0.162
	*p*	0.444	0.687	0.321	0.199	0.547	0.863	0.293
**miR-29a-3p**	r	−0.129	−0.047	−0.008	0.222	0.031	−0.027	−0.210
	*p*	0.414	0.762	0.959	0.153	0.841	0.863	0.171
**miR-29b-3p**	r	**−0.385**	0.038	0.079	0.065	−0.163	−0.056	−0.149
	*p*	**0.013**	0.810	0.621	0.685	0.304	0.724	0.340
**miR-29c-3p**	r	**−0.360**	−0.099	0.053	0.115	0.047	0.011	−0.186
	*p*	**0.019**	0.522	0.735	0.461	0.764	0.945	0.226
**miR-93-5p**	r	−0.174	−0.188	**−0.461**	−0.146	−0.134	0.081	0.174
	*p*	0.271	0.222	**0.002**	0.349	0.393	0.605	0.258
**miR-99a-5p**	r	−0.231	−0.016	−0.261	−0.031	−0.181	−0.022	0.066
	*p*	0.142	0.920	0.091	0.843	0.246	0.890	0.670
**miR-146a-5p**	r	−0.170	−0.130	**−0.510**	−0.075	0.035	−0.043	0.030
	*p*	0.282	0.399	**<0.001**	0.631	0.822	0.783	0.847
**miR-195-5p**	r	−0.162	−0.005	−0.073	0.129	−0.295	−0.011	−0.078
	*p*	0.306	0.973	0.640	0.411	0.055	0.945	0.615

**Table 5:** Correlation between circulating microRNAs and bone disease or spinal osteolytic lesions in multiple myeloma patients. For each miRNA, the Spearman’s correlation coefficient (r) and significance level (*p*-value) are shown. Statistically significant correlations are highlighted in blue.

		Bone Disease	Regions of the Spine			
			Cervical	Thoracic	Lumbar	Sacrum
**miR-16-5p**	r	−0.147	0.118	0.127	−0.163	0.175
	*p*	0.341	0.456	0.415	0.303	0.267
**miR-20a-5p**	r	−0.073	0.076	0.146	−0.119	0.155
	*p*	0.640	0.633	0.349	0.453	0.327
**miR-21-5p**	r	0.057	−0.270	−0.007	0.202	0.060
	*p*	0.714	0.084	0.962	0.199	0.706
**miR-29a-3p**	r	−0.041	−0.112	−0.022	−0.103	0.270
	*p*	0.791	0.479	0.886	0.515	0.084
**miR-29b-3p**	r	−0.135	**−0.340**	**−0.304**	−0.205	0.153
	*p*	0.389	**0.030**	**0.050**	0.198	0.338
**miR-29c-3p**	r	**−0.308**	−0.215	−0.191	−0.226	−0.125
	*p*	**0.042**	0.171	0.219	0.150	0.430
**miR-93-5p**	r	−0.100	−0.185	−0.079	0.087	0.115
	*p*	0.518	0.241	0.616	0.582	0.468
**miR-99a-5p**	r	−0.151	−0.215	−0.120	−0.008	0.285
	*p*	0.328	0.171	0.444	0.960	0.067
**miR-146a-5p**	r	−0.226	−0.112	−0.015	0.032	−0.025
	*p*	0.141	0.479	0.924	0.842	0.875
**miR-195-5p**	r	−0.080	−0.143	**−0.307**	0.000	0.095
	*p*	0.604	0.368	**0.045**	1.000	0.549

## Data Availability

The data presented in this study are available on request from the corresponding author. The data are not publicly available due to ethical reasons.

## Supplementary Materials

The following are available online at https://www.mdpi.com/article/10.3390/cancers13215258/s1, Table S1: Receiver Operating Characteristic (ROC) curve analysis for circulating miR-16-5p, miR-20a-5p and miR-21-5p, or their combination (miRNA panel), when analyzing MM patients versus healthy controls. Table S2: Statistical differences in miRNA levels between multiple myeloma patients with and without bone lesions.
