# Supplementary material for: Circulating microRNAs Correlate with Multiple Myeloma and Skeletal Osteolytic Lesions
Source: Cancers (Basel). 2021 Oct 20;13(21):5258. doi: 10.3390/cancers13215258 (PMC8582565; doi:10.3390/cancers13215258)
Supplement: Supplementary file 1 [file cancers-13-05258-s001.zip › cancers-1342644-supplementary.pdf]

cancers

MDPI

Supplementary Material

Circulating microRNAs Correlate with Multiple Myeloma and Skeletal Osteolytic Lesions

Sara Reis Moura, Hugo Abreu, Carla Cunha, Cláudia Ribeiro-Machado, Carla Oliveira, Mario Adolfo Barbosa, Herlander Marques and Maria Inês Almeida

Table S1. Receiver Operating Characteristic (ROC) curve analysis for circulating miR-16-5p, miR-20a-5p and miR-21-5p, or their combination (miRNA panel), when analyzing MM patients versus healthy controls.

	Healthy vs. MM							
	**AUC**	**SD**	***p*-value**	**CI 95 %**		**Cut-off**	**Sensivity**	**Specificity**
				**Lower**	**Upper**	**(Youden's index)**	**(%)**	**(%)**
**miR-16-5p**	0.665	0.061	0.011	0.5458	0.7843	4.417	78.72	51.43
**miR-20a-5p**	0.707	0.057	0.001	0.5961	0.8179	0.01145	36.17	97.14
**miR-21-5p**	0.654	0.060	0.018	0.5365	0.7717	0.002328	57.45	71.43
**miRNA panel**	0.825	0.047	<0.0001	0.7333	0.9166	0.5434	78.72	80.00

MM—Multiple Myeloma; AUC—Area Under the Curve; SD—Standard deviation; CI—Confidence Interval

Table S2. Statistical differences in miRNA levels between multiple myeloma patients with and without bone lesions. Statistically significant differences are highlighted in bold (Mann–Whitney test, * $p < 0.05$).

**miRNA**	***p*-value**
miR-16-5p	0.3412
miR-20a-5p	0.6433
miR-21-5p	0.7188
miR-29a-3p	0.7970
miR-29b-3p	0.3901
**miR-29c-3p**	**0.0448 ***
miR-93-5p	0.5202
miR-99a-5p	0.3283
miR-146a-5p	0.1426
miR-195-5p	0.6069
